# Supplementary material for: Bioconversion of a Dairy By-Product (Scotta) into Mannitol-Stabilized Violacein via Janthinobacterium lividum Fermentation
Source: Microorganisms. 2025 Sep 11;13(9):2125. doi: 10.3390/microorganisms13092125 (PMC12472305; doi:10.3390/microorganisms13092125)
Supplement: Supplementary file 1 [file microorganisms-13-02125-s001.zip › microorganisms-3810283-supplementary.pdf]

GEN III Microplate Map

A-1 Negative Control	A-2 Dextrin	A-3 D-Maltose	A-4 D-Trehalose	A-5 D-Cellobiose	A-6 Gentiobiose	A-7 Sucrose	A-8 D-Turanose	A-9 Stachyose	A-10 Positive Control	A-11 pH 6	A-12 pH 5
B-1 D-Raffinose	B-2 α-D-Lactose	B-3 D-Melibiose	B-4 β-Methyl-D-Glucoside	B-5 D-Salicin	B-6 N-Acetyl-D-Glucosamine	B-7 N-Acetyl-β-D-Mannosamine	B-8 N-Acetyl-D-Galactosamine	B-9 N-Acetyl Neuraminic Acid	B-10 1% NaCl	B-11 4% NaCl	B-12 8% NaCl
C-1 α-D-Glucose	C-2 D-Mannose	C-3 D-Fructose	C-4 D-Galactose	C-5 3-Methyl Glucose	C-6 D-Fucose	C-7 L-Fucose	C-8 L-Rhamnose	C-9 Inosine	C-10 1% Sodium Lactate	C-11 Fusidic Acid	C-12 D-Serine
D-1 D-Sorbitol	D-2 D-Mannitol	D-3 D-Arabitol	D-4 myo-Inositol	D-5 Glycerol	D-6 D-Glucose-6-PO4	D-7 D-Fructose-6-PO4	D-8 D-Aspartic Acid	D-9 D-Serine	D-10 Macrolide	D-11 Rifamycin SV	D-12 Minocycline
E-1 Gelatin	E-2 Glycyl-L-Proline	E-3 L-Alanine	E-4 L-Arginine	E-5 L-Aspartic Acid	E-6 L-Glutamic Acid	E-7 L-Histidine	E-8 L-Pyroglutamic Acid	E-9 L-Serine	E-10 Lincomycin	E-11 Guanidine HCl	E-12 Niaproof 4
F-1 Pectin	F-2 D-Galacturonic Acid	F-3 L-Galactonic Acid Lactone	F-4 D-Gluconic Acid	F-5 D-Glucuronic Acid	F-6 Glucuronamide	F-7 Mucic Acid	F-8 Quinic Acid	F-9 D-Saccharic Acid	F-10 Vancomycin	F-11 Tetrazolium Violet	F-12 Tetrazolium Blue
G-1 p-Hydroxy-Phenylacetic Acid	G-2 Methyl Pyruvate	G-3 D-Lactic Acid Methyl Ester	G-4 L-Lactic Acid	G-5 Citric Acid	G-6 α-Keto-Glutaric Acid	G-7 D-Malic Acid	G-8 L-Malic Acid	G-9 Bromo-Succinic Acid	G-10 Nalidixic Acid	G-11 Lithium Chloride	G-12 Potassium Tellurite
H-1 Tween 40	H-2 γ-Amino-Butyric Acid	H-3 α-Hydroxy-Butyric Acid	H-4 β-Hydroxy-D,L-Butyric Acid	H-5 α-Keto-Butyric Acid	H-6 Acetoacetic Acid	H-7 Propionic Acid	H-8 Acetic Acid	H-9 Formic Acid	H-10 Aztreonam	H-11 Sodium Butyrate	H-12 Sodium Bromate

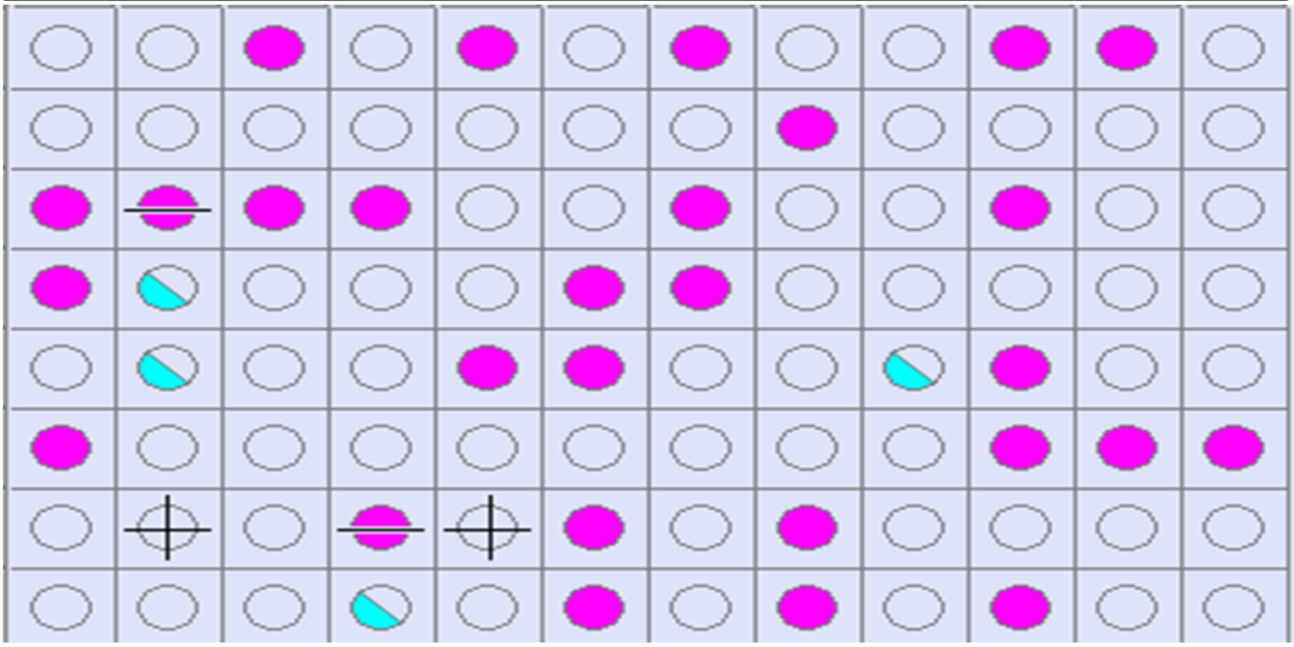

Figure S 1. GEN III microplate biochemical profile of J. lividum DSM1522

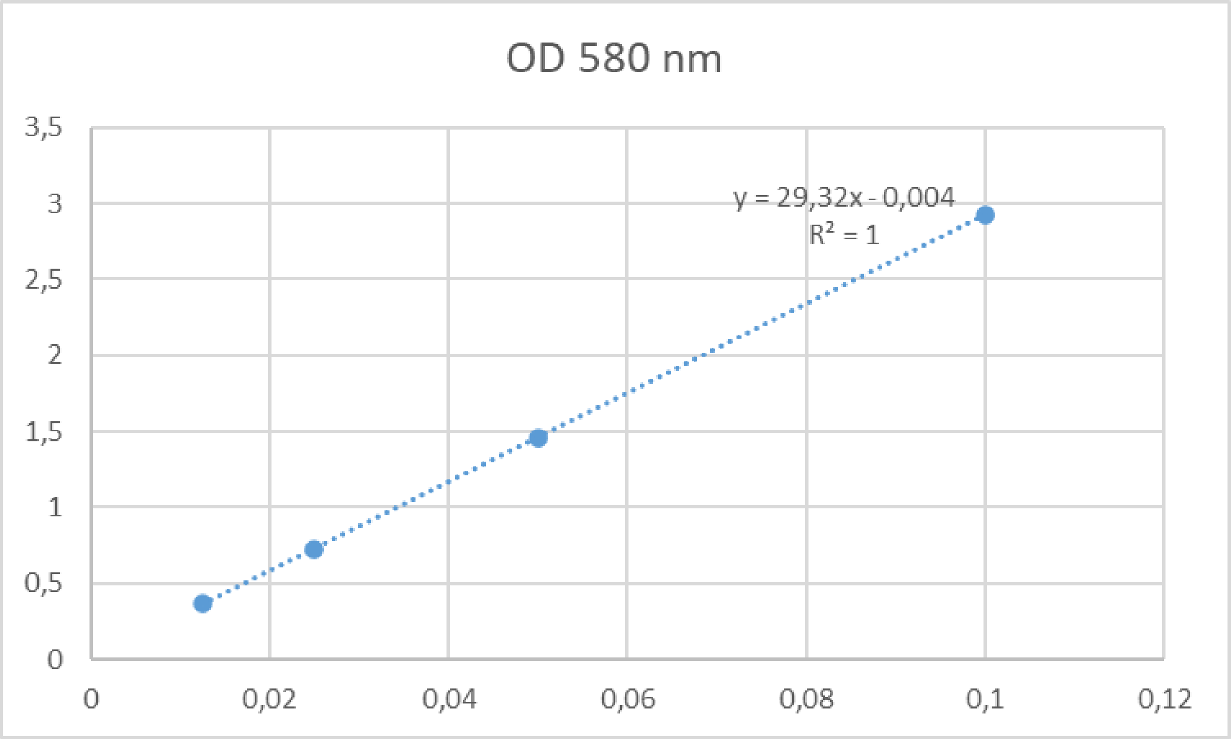

Figure S 2. Calibration curve for relationship between violacein standard (Sigma-Aldrich, V9389) and optical density (580 nm)

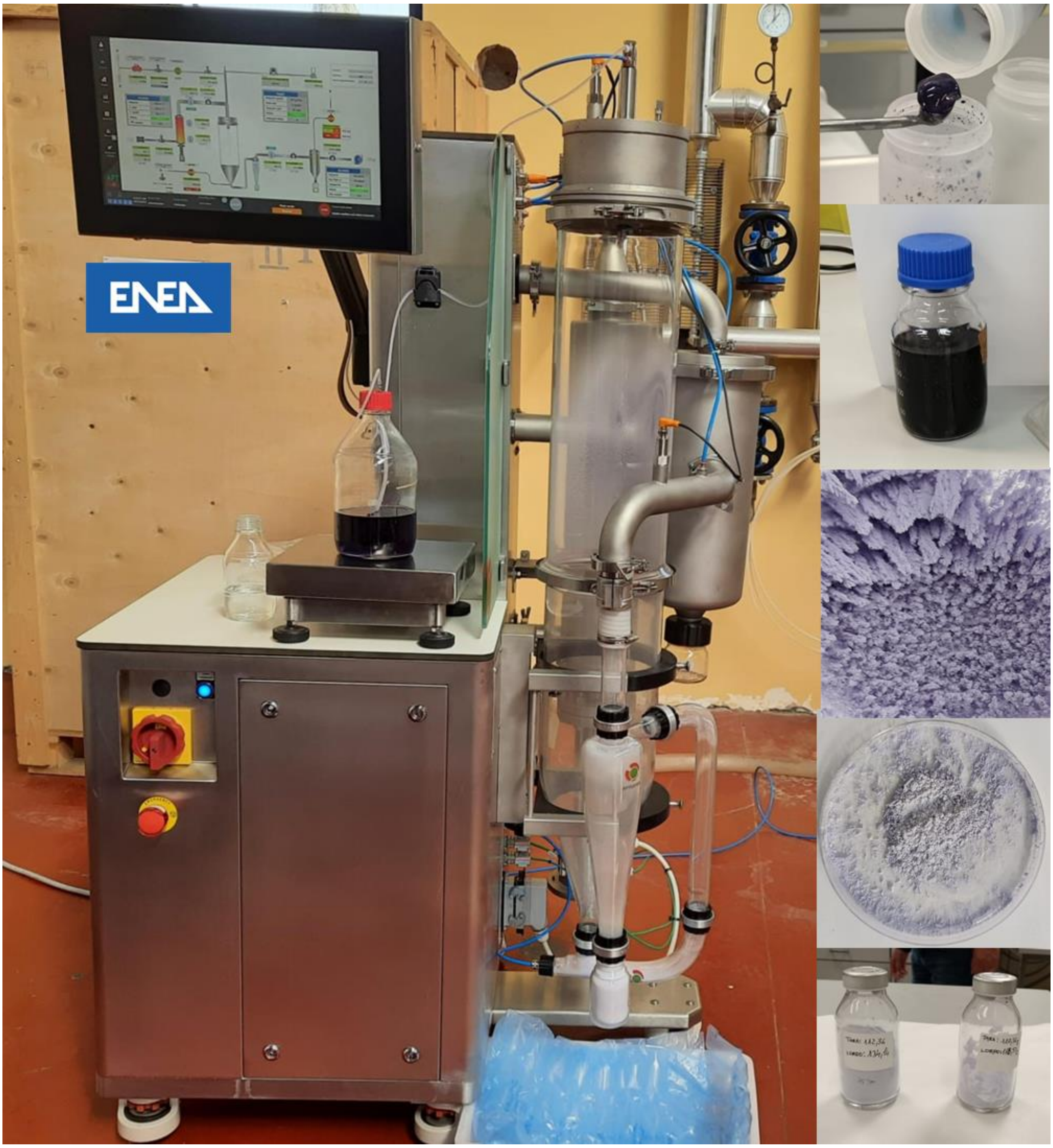

Figure S 3. Lab-scale APT-2.0 spray dryer (left) and illustration of dryer process (right) from top to bottom: biomass recovery, violacein extraction, inside cyclone surface, detail of recovered powder and powder under nitrogen in crown cap bottles
